# Discrepancies between upper GI symptoms described by those who have them and their identification by conventional medical terminology: a survey of sufferers in four countries

**DOI:** 10.1097/MEG.0000000000000565

**Published:** 2016-03-09

**Authors:** Robert C. Heading, Edward C.M. Thomas, Phil Sandy, Gary Smith, Ronnie Fass, Pali S. Hungin

**Affiliations:** a School of Medicine, Pharmacy and Health, Durham University, Durham; b Category Development Organisation, Reckitt Benckiser Plc, Slough, UK; c Chief Research Officer, Winkle BV, Keizersgracht , Amsterdam, The Netherlands; d Department of Gastroenterology, Case Western Reserve University, Cleveland, Ohio, USA

**Keywords:** dyspepsia, gastroesophageal reflux, heartburn, humans, questionnaires, symptoms, upper gastrointestinal tract, vocabulary

## Abstract

**Objective:**

The objective of this study was to develop a self-administered questionnaire for upper gastrointestinal (GI) symptoms using lay vocabulary uninfluenced by established medical terminology or concepts and to conduct a survey of symptom occurrence among sufferers in four countries.

**Methods:**

The questionnaire was designed by integrating information gained from the vocabulary used by 38 upper GI symptom sufferers. There was no medical input to its development. The questionnaire was then used, after appropriate translation, in Brazil, Russia, the UK and the USA. Details of 10 659 symptom episodes were obtained from 2665 individuals.

**Results:**

Nine symptoms described in lay vocabulary were identified during questionnaire development. Of these, one corresponded to regurgitation, whereas two that were distinguished by survey participants might both be interpreted as heartburn. One chest symptom for which a corresponding medical term was uncertain occurred in ∼30% of the respondents. Five different ‘stomach’ or abdominal symptoms were identified. The predominant symptom and the pattern of concurrent symptoms often varied from one symptom episode to another. Use of the terms ‘heartburn’, ‘reflux’, ‘indigestion’ and ‘burning stomach’ to describe symptoms varied between countries.

**Conclusion:**

Some common upper GI symptoms described by those who suffer them have no clear counterpart in conventional medical terminology. Inadequacy of the conventional terminology in this respect deserves attention, first, to characterize it fully, and thereafter to construct enquiry that delivers more precise symptom identification. Our results suggest that improvement may require the use of vocabulary of individuals suffering the symptoms without imposing conformity with established symptom concepts.

## Abstract 2

Supplemental Digital Content is available in the text.

## Introduction

Most studies on upper gastrointestinal (GI) symptom occurrence are founded on the use of questionnaires. Various methodologies have been used to develop them, but patient input into their development has become the norm 1–8. For example, the Glasgow Dyspepsia Questionnaire 1, the Global Overall Symptom scale 2, the Leeds Dyspepsia Questionnaire 3, the Nepean Dyspepsia Index 4, the Reflux Disease Questionnaire 5, ReQuest 6, and the REFLUX questionnaire 7 have all been designed utilizing information obtained from patients. Self-administered questionnaires have been favoured in recent years, with the involvement of focus groups to help formulate the wording of the questions such that they will be readily understood by patients 4–7. This ‘patient-friendly’ wording is then reconciled with established medical terminology, which is the basis of subsequent analysis. To our knowledge, no survey has yet used an upper GI symptom questionnaire constructed using layperson-based language without reference to the established medical symptom vocabulary.

Our study aimed to create a self-administered questionnaire for upper GI symptoms using layperson vocabulary without imposition of medical terminology or concepts, and to use it in a survey of symptom occurrence among sufferers in four countries.

## Methods

### Survey content

The survey and an online diary were developed and undertaken, and the responses were collated by two specialist market research companies (Winkle BV, Keizersgracht, Amsterdam, The Netherlands and Msi-aci BV, Joop Geesinkweg, Amsterdam, The Netherlands).

Lay market research agency screening identified participants (eight in Brazil and 10 each in Russia, the UK and the USA) aged between 18 and 64 years who had experienced upper GI symptoms within the previous 3 months. Moderators then conducted a series of qualitative one on one interviews in which they asked these participants to describe their symptoms in their own words, describing both the physical sensation they experienced and its location. Multiple symptoms, sometimes concurrent, were described in many instances. All these descriptions were then collated and used to draft questions in lay language (i.e. without using any medical terminology) for the questionnaire and diary. Symptoms that were infrequent among the participants interviewed were not taken into consideration for the questionnaire.

The questions were first drafted in English and then translated by a professional translation agency into the local languages. The drafts were then sent back to the moderators who had conducted the interviews for review and any revision necessary to ensure that the translations accorded with their original interview findings.

### Screening and extended survey

Members of a market research panel were invited by email to complete the questionnaire entitled ‘New Survey about Health Issues’. The invitees, aged between 18 and 64 years, in Brazil, Russia, the UK and the USA were drawn at random from the market research panel. Panel membership required that the individual be responsible, either mainly or jointly, for shopping for medicines/medications.

**Fig. 1:**
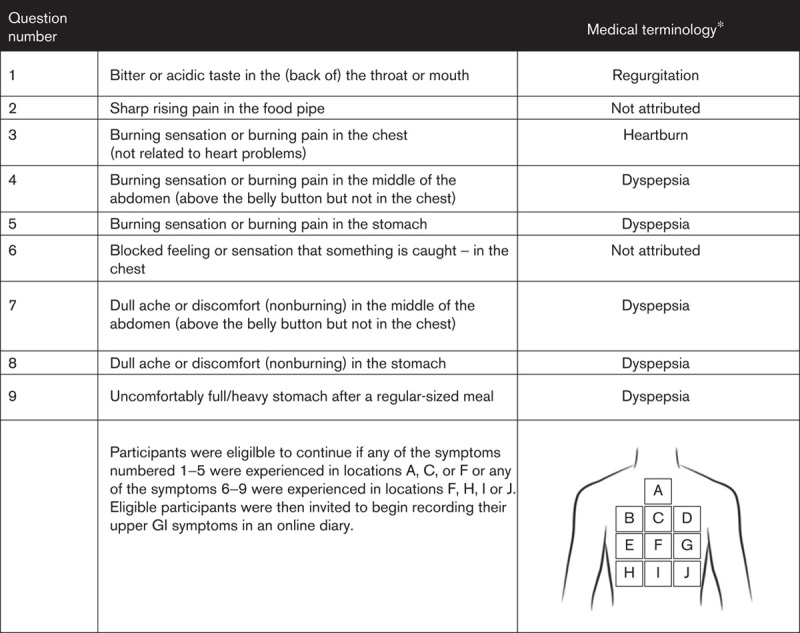
Eligibility criteria. Participants were asked whether they had experienced any of these symptoms in the past 3 months. If so, they were asked ‘Where in your body did you feel this symptom was located?’ and to respond with reference to the diagram. *See text for explanation of proposed medical terminology. GI, gastrointestinal.

This initial screening survey consisted of a demographic questionnaire and questions to establish whether the participant had experienced any of the specified upper GI symptoms in the appropriate part of the body within the last 3 months (Fig. 1). Individuals answering positively were allowed access to an extended survey, which asked about the last symptom episode in more depth, including a question about what term they would use to describe their ailment to a doctor or a friend.

A target respondent sample size of 450 per country was set. Allowing for a drop-out rate of 50% between the survey and diary stages, the target was ∼900 per country for the initial survey.

### Online diary

Participants who entered the diary phase were emailed a link each day for up to 6 weeks between June and August 2010. They were asked to indicate whether they had experienced any of the listed symptoms that day in the labelled areas of their bodies (symptom locations shown in Fig. 1; see text, Supplemental digital content 1, *http://links.lww.com/EJGH/A70* for the online diary questions). The participants were then asked more detailed questions on up to seven symptom episodes (occurrence of symptoms) experienced on different days during the 6-week period. The information requested included identification of the predominant (most intense) symptom on each occasion, rating its severity as mild, average or severe, and indicating its duration, timing and location (see text, Supplemental digital content 1, *http://links.lww.com/EJGH/A70*). Answers to other questions about psychological and emotional responses to the symptoms, medications and any action taken in an attempt to gain relief have not been reported in this paper.

### Statistical analysis of symptoms

Data were analyzed using SAS software (SAS Institute Inc., Cary, North Carolina, USA) and descriptive statistics compiled according to the following populations: (a) survey responders; (b) survey and diary responders; (c) survey and diary responders with more than one diary episode; (d) all episodes in diary. Analysis was carried out with all nine symptoms as reported by responders and, subsequently, with symptoms partly grouped according to medical terminology. For this latter purpose, positive responses to question 1 (Fig. 1) were considered to be regurgitation, to question 3 to be heartburn and to questions 4, 5, 7, 8 and 9 to be dyspepsia. Owing to uncertainty about the appropriate medical terms for symptoms represented by questions 2 and 6, no conventional medical term was applied to them, and they are abbreviated hereafter as ‘sharp rising pain: food pipe’ and ‘blocked feeling: chest’, respectively.

The binomial outcomes collected at the participant level were compared between sex and age group (<45 years, ≥45 years) using a *χ*^2^-test.

The ordinal outcomes collected at the participant level were analyzed using a logistic regression model, with country as a fixed effect. Pairwise comparisons between countries were carried out from these models.

The binomial or ordinal outcomes collected per symptom episode across online diary participants were analyzed using logistic regression models with either sex, age group (<45 years, ≥45 years) or country included as fixed effects and participant as a random effect. Pairwise comparisons between countries were carried out using these models.

## Results

### Survey and diary completion

The screening survey identified a total of 5158 participants with the specified upper GI symptoms in the appropriate part of the body within the previous 3 months. Of these, 2665 provided diary responses related to 10 659 symptom episodes. Demographic data are presented in Table 1.

**Table 1:**
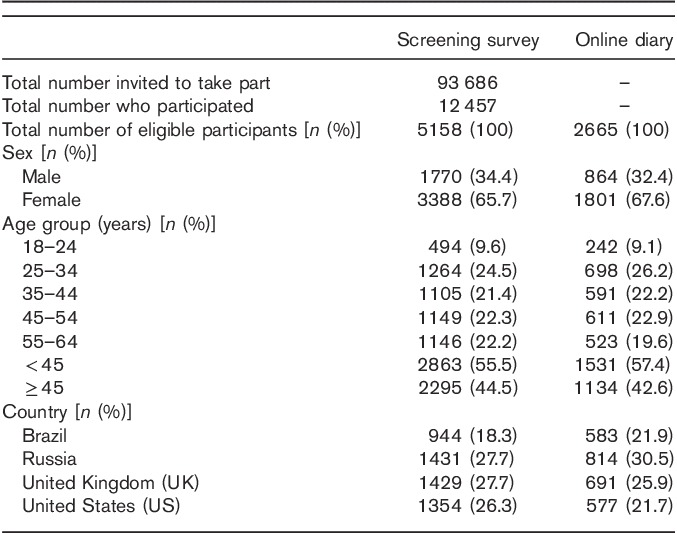
Demographic data of the eligible respondents of the screening survey and diary completers

### Incidence of symptoms as described in the online diary

Table 2 shows the symptoms reported by the participants. ‘Uncomfortably full/heavy stomach after a regular sized meal’ and ‘bitter or acidic taste in the back of the throat or mouth’ (regurgitation) were the most frequent; ‘burning sensation in the middle of the abdomen’ was least frequently reported. ‘Blocked feeling or sensation that something is caught in the chest’ and ‘bitter or acidic taste’ occurred with similar frequencies in all countries. Some other symptoms such as burning sensation in the chest and burning sensation in the stomach showed statistically significant differences between countries, but the relative frequencies of the nine symptoms were broadly similar in all. Most symptoms were equally frequent in male and female participants, but they were more frequent in the older than in the younger age group.

**Table 2:**
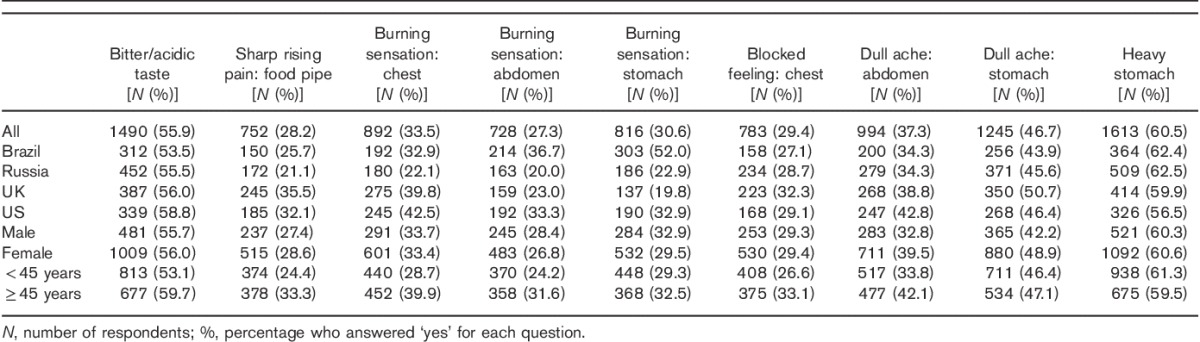
Incidence of online diary reported symptoms

### Predominant symptoms

#### Symptom predominance and severity

When the five symptoms comprising ‘dyspepsia’ (see Fig. 1) were considered as one entity, it was the most commonly reported predominant symptom, being identified as such by 2121 (79.6%) participants during at least one episode. ‘Bitter/acidic taste’ (regurgitation) was reported as the predominant symptom at least once by 1158 (43.5%) participants, ‘burning sensation: chest’ (heartburn) by 525 (19.7%), ‘blocked feeling: chest’ by 443 (16.6%) and ‘sharp rising pain: food pipe’ by 385 (14.4%) participants.

The frequency with which each of the five symptoms was reported as predominant varied by country (*P*≤0.0022; see Figure, Supplemental digital content 2, *http://links.lww.com/EJGH/A71*). Notable differences were observed for ‘blocked feeling: chest’, which was most frequent in Russia (164, 20.1%) and least frequent in Brazil (60, 10.3%), and ‘burning sensation: chest’ had a higher prevalence in the UK (187; 27.1%) and the USA (146, 25.3%) compared with Brazil (78, 13.4%) and Russia (114, 14.0%).

**Fig. 2:**
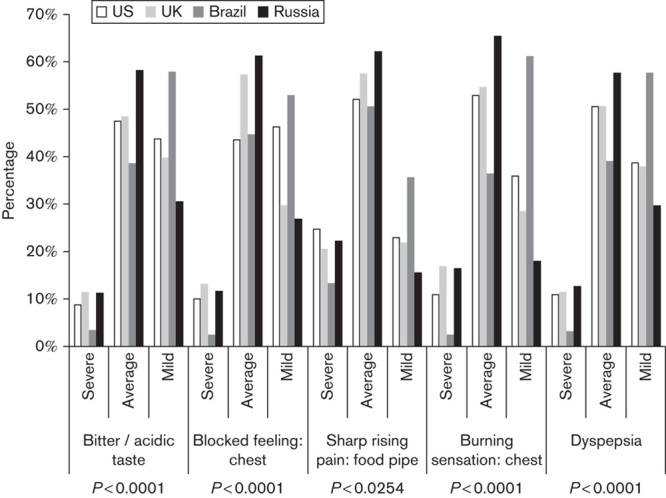
Severity of predominant symptom by country. *P*-values relate to variability of predominant symptoms across episodes reported by individual participants (ordinal logistic regression with random participant effect).

Reported severity of the symptoms varied by country (Fig. 2). Overall, the predominant symptoms were mostly classed as ‘average’ in severity (51.2%); 38% were classed ‘mild’ and 10.9% as ‘severe’. Fewer participants in Brazil reported their predominant symptom as severe and more described it as mild compared with the USA, UK and Russian participants (*P*<0.0001 for all comparisons).

**Fig. 3:**
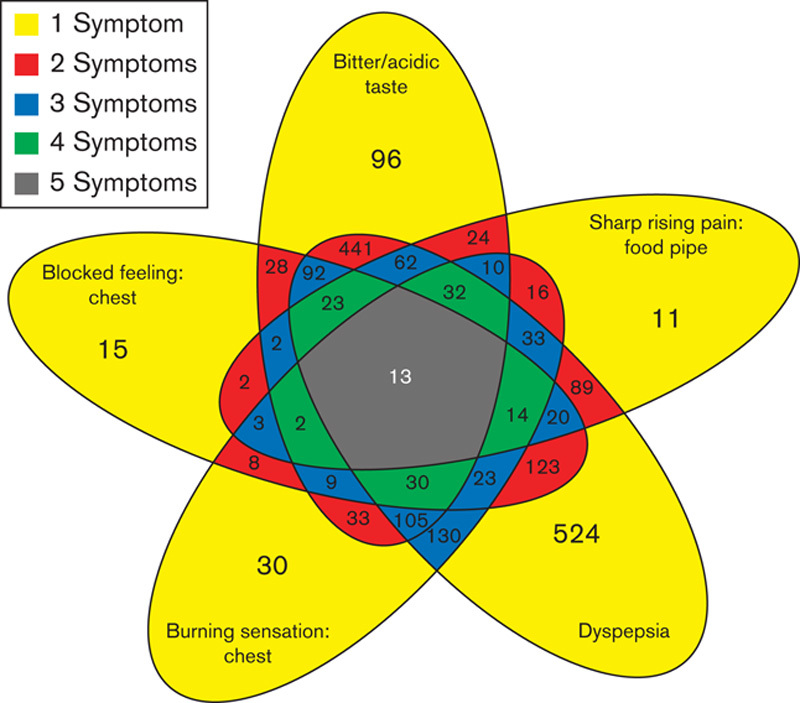
Predominant symptoms reported by respondents across all their symptom episodes.

The predominant symptom varied in 67% of those who reported more than one symptom episode. Two different predominant symptoms were reported on different occasions by 894 (44%) participants, three different symptoms by 359 (18%), four different symptoms by 101 (5%) and five different predominant symptoms by 13 (0.6%) participants (Fig. 3).

#### Predominant symptom timing

**Fig. 4:**
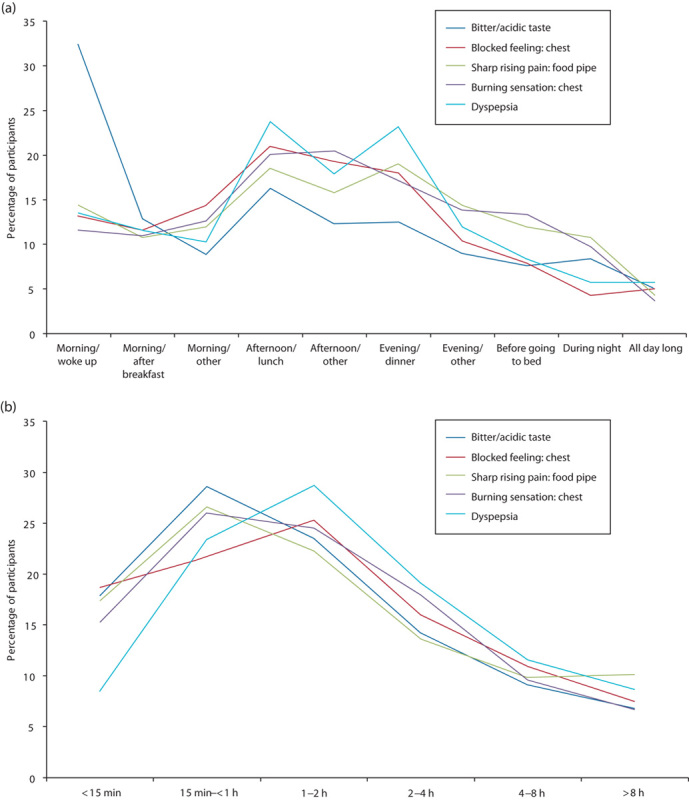
(a) Percentage of study participants who experienced the specified symptoms at their strongest at the stated time, and (b) percentage of study participants who experienced the specified predominant symptoms for the stated durations.

Participants also recorded the time of day when their predominant symptom was at its strongest (Fig. 4a). Overall, ‘bitter/acidic taste’ was most commonly reported in the morning upon waking (32.4%), whereas postprandial periods were particularly associated with the occurrence of other symptoms. ‘Burning sensation: chest’ was most commonly reported in the afternoon after lunch (20.1%) and at other times in the afternoon (20.5%). ‘Sharp rising pain: food pipe’ was most commonly reported after lunch (18.5%) and dinner (19.0%), with a combined after lunch/dinner prevalence of 37.6%. ‘Dyspepsia’ was most common after lunch (23.8%) and dinner (23.2%), with a combined after lunch/dinner prevalence of 47.0%.

An effect of country on the prevalence of ‘bitter/acidic taste’ in the morning was observed (*P*<0.0001), with Brazil and Russia reporting an approximately two-fold greater prevalence in this symptom at this time point (41.8 and 44.7%, respectively) than the USA and UK (21.5 and 21.0%, respectively). Pairwise comparisons for the USA or UK versus Brazil or Russia were all highly significant (*P*<0.0001). The prevalence of ‘sharp rising pain: food pipe’ after lunch/dinner also varied by country, with Russia having a significantly lower prevalence (23.7%) compared with the USA (37.0%, *P*=0.0296) and the UK (45.1%, *P*=0.0008), although not significant when compared with Brazil (37.3%, *P*=0.0554).

#### Predominant symptom duration

The reported durations of the predominant symptoms are shown in Fig. 4b. ‘Bitter or acidic taste in the back of the throat or mouth’, ‘sharp rising pain in the food pipe’ and ‘burning sensation or burning pain in the chest’ most commonly lasted between 15 min and 1 h (28.6, 26.6 and 26.0%, respectively). ‘Blocked feeling or sensation that something is caught in the chest’ and ‘dyspepsia’ most commonly lasted between 1 and 2 h (25.3 and 28.7%, respectively).

Overall, there were differences in the duration of predominant symptoms between all countries except the USA and the UK (*P*<0.0001). Sex also demonstrated differences (*P*<0.0001). There were no differences between age groups (see Figures, Supplemental digital content 3, *http://links.lww.com/EJGH/A72*, Supplemental digital content 4, *http://links.lww.com/EJGH/A73*, and Supplemental digital content 5, *http://links.lww.com/EJGH/A74* for duration of predominant symptoms by country, age and sex, respectively).

### Symptom concurrence

**Fig. 5:**
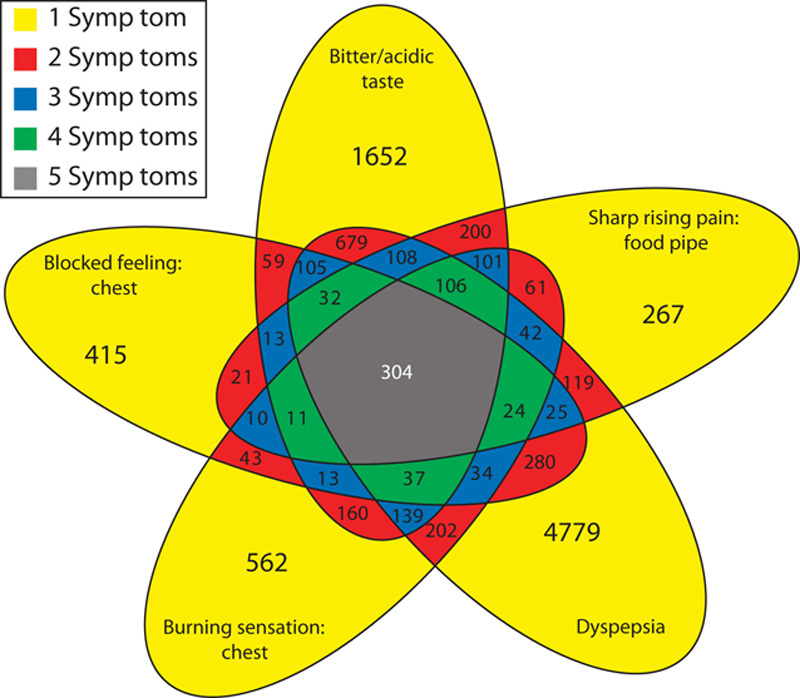
Frequency of symptoms reported concurrently across 10 603 symptom episodes.

Across all episodes, multiple symptoms were reported on 28% of 10 603 occasions (Fig. 5). The discrepancy with the 10 659 symptom occasions shown in Fig. 2 arose from failure to complete Section A of the diary (See text, Supplemental digital content 1, *http://links.lww.com/EJGH/A70*) on 56 occasions. Dyspepsia accounted for most instances in which a single symptom was reported (45% of all episodes), with ‘bitter or acidic taste in the throat or mouth’, ‘blocked feeling or sensation that something is caught in the chest’, ‘burning sensation or burning pain in the chest’ and ‘sharp rising pain in the food pipe’ being the single symptom in 15.6, 3.9, 5.3 and 2.5% of episodes, respectively. Overall, two concurrent symptoms were reported on 17.2% of occasions and the rates of simultaneously suffering three, four and five concurrent symptoms were 5.6, 2.0 and 2.9%, respectively (Fig. 5).

Particular attention was paid to the sharp rising pain in the food pipe and the burning sensation or pain in the chest in relation to the question of whether one or both should be considered to correspond to heartburn. There were 1975 occasions on which respondents reported experiencing either the sharp rising pain in the food pipe or the burning sensation or pain in the chest and a further 659 occasions (in 348 individuals) on which the two were reported concurrently (Fig. 5). In addition to the occasions on which the two symptoms occurred concurrently, 58% of these individuals reported other occasions on which they experienced either the sharp rising pain in the food pipe or the burning sensation or pain in the chest exclusively.

### Correlation of patient language with symptom experience

The screening survey asked participants what term they would use to describe their ailment. A high proportion (544; 56.8%) of people from Russia, who experienced ‘bitter or acidic taste in the back of the throat or mouth’, called it ‘heartburn’, with only 145 (15.2%) describing their symptom as reflux/acid reflux. ‘Heartburn’ was also the most common term used to describe bitter/acidic taste in Brazil (238; 38.0%) compared with reflux/acid reflux, preferred by 187 (29.9%) of subjects. Interestingly, 92 (14.7%) participants in Brazil described ‘bitter/acidic taste’ as ‘burning stomach’, compared with 1.9% of respondents across the other countries. By contrast, reflux/acid reflux was the most common descriptor for the bitter/acidic taste in the back of the throat or mouth in the USA and the UK (448, 52.6% and 431, 48.4%, respectively).

The majority of participants in the UK and the USA called their burning sensation in the chest ‘heartburn’ (397, 52.4% and 474, 51.9%, respectively). However, only 95 (22.1%) and 176 (29.1%) participants in Brazil and Russia called this symptom heartburn; it was more commonly described as ‘burning stomach’ (89, 20.7%) in Brazil, ‘indigestion’ (98, 16.2%) in Russia or ‘other’ (111, 25.9% Brazil and 105, 17.4% Russia). The term ‘indigestion’ was also used for ‘burning sensation in the chest’ by 263 (28.8%) UK participants.

## Discussion

Uncertainty about translating a patient’s description of symptoms into established medical terminology is familiar to many physicians, who acknowledge that conventional terminology may not capture some aspects of symptom perception that patients describe. Nevertheless, we are not aware of any previous attempt at systematic creation of an upper GI symptom questionnaire from sufferers’ own vocabulary without reference to conventional medical concepts or terminology. Our survey results describe the occurrence and patterns of upper GI symptoms in 10 659 symptom episodes.

Matching the lay vocabulary of our questionnaire with medical terminology revealed substantial disconnect. Whereas ‘bitter or acidic taste in the back of the throat or mouth’ may be interpreted as ‘regurgitation’, not least because it was prominent on wakening in the morning 9, equating ‘burning sensation or burning pain in the chest’ with heartburn implies that heartburn occurred only about two-thirds as often as regurgitation in this population. Burning chest pain or discomfort is widely taken to be a description of heartburn, although Carlsson *et al.* 10 advocated a definition in which the moving quality of the feeling was recognized (a burning feeling rising from the stomach or lower chest up towards the neck) and asserted that this served to identify heartburn responsive to acid suppressing medication. If ‘burning sensation or burning pain in the chest’ and ‘sharp rising pain in the food pipe’ are considered to be alternative descriptions of heartburn, combining the two implies that heartburn occurred more frequently than regurgitation among our respondents. However, the fact that 58% of individuals who reported having the two symptoms concurrently on some occasions also reported having one without the other on other occasions implies they are not simply alternative descriptions of the same sensation. Consequently, use of the term ‘heartburn’ to denote both does not accurately represent the symptoms being recognized by sufferers.

The symptom ‘blocked feeling or sensation that something is caught in the chest’ is also problematic. At first, it might be thought to correspond to dysphagia, but sufferers commonly reported a duration of 1–2 h, which seemingly points against a direct relationship with swallowing. No specific enquiry about swallowing was incorporated in the questionnaire, and the online methodology of this study did not allow further interrogation to characterize the nature of the symptom further. Nevertheless, ‘blocked feeling or sensation that something is caught in the chest’ was reported in almost one-third of respondents in all four countries and, of course, was derived from the original interviews with those experiencing the symptom. It is therefore hard to refute that this wording describes a sensation experienced by a significant proportion of individuals, but we cannot propose an obvious medical counterpart.

Unsurprisingly, dyspepsia was the most common symptom reported in all countries when the five symptoms perceived in the ‘stomach’ or abdomen were grouped together. Our results add to the existing evidence of overlap between reflux and dyspepsia symptoms 11–13, with 28% of respondents experiencing at least two symptoms concurrently on any one occasion. This corresponds closely to the findings of a community survey in which coexisting dyspepsia and reflux symptoms occurred in 24% of those reporting symptoms 12. The overlap between reflux disease and dyspepsia and whether to consider them as a single entity 14 are key areas of debate that are highly relevant to the challenge of accurate clinical diagnosis.

It is obvious that use of the single term ‘dyspepsia’ does not respect the fact that our study participants were describing five ‘stomach’ and abdominal symptoms that they considered could be distinguished. Such conflation of symptoms itself raises potential problems for precision of diagnosis and choice of treatment. Acknowledging this, the Rome III classification of functional dyspepsia refined earlier definitions by introducing a distinction between postprandial distress and epigastric pain syndromes. A form of enquiry to identify the former has been proposed 15, but these two variants of functional dyspepsia often occur concurrently, prompting some to suggest that this classification is inherently unsatisfactory 16. However, better identification of symptoms gained from more detailed enquiry may be helpful 17,18. In addition, ‘heartburn’ is said to occur commonly in individuals with functional dyspepsia 14. Our results have shown that besides using the word ‘dyspepsia’ to describe five different symptoms, the single word ‘heartburn’ cannot properly represent both ‘sharp rising pain in the food pipe’ and ‘burning sensation or pain in the chest’. Thus, neither dyspepsia nor heartburn is a precise term. Greater precision is required for both if diagnosis and classification of upper GI disorders are to be improved.

Another aspect of our results with potential relevance to clinical practice is the observation that two-thirds of participants reported different predominant symptoms on different occasions. To our knowledge, the magnitude of this variability has not been demonstrated previously. Some years ago, it was reported that a diagnosis of reflux disease was likely to be correct if heartburn or regurgitation was a clearly predominant symptom 19. Subsequent consensus statements pointed out that although predominant heartburn was thought to permit a diagnosis of gastro-oesophageal reflux disease in 75–80% of patients, this belief was based on clinical opinion rather than further evidence 20,21. More recently, guidelines have simply advised that ‘typical symptoms of heartburn and regurgitation’ justify a presumptive gastro-oesophageal reflux disease diagnosis 22. Our findings show that in many participants both the predominant symptom and the pattern of concurrent symptoms vary from one symptom episode to the next. Only one-third of individuals had one symptom that was consistently predominant.

Apart from ‘bitter or acidic taste in the back of the throat or mouth’, which was most common on waking up in the morning, the majority of predominant symptoms occurred mainly after meals. The pattern of predominant nocturnal symptoms differed from that of daytime symptoms. The prevalence of nocturnal symptoms in our participants was low compared with some reports 23, although not all investigators found nocturnal symptoms to be common 24. Our results were almost certainly influenced by inclusion of individuals with relatively mild symptoms, and it is also possible that our questionnaire did not reliably identify symptoms occurring during the ‘recumbent awake’ period, said to be especially important in reflux disease 25.

National differences in the medical terms that participants thought to be appropriate to describe their symptoms were evident in our results. Most notably, the symptom identified in all four countries as ‘bitter or acidic taste in the back of the throat or mouth’ was termed ‘heartburn’ by many Russian participants and some Brazilian participants, whereas in the USA and UK, it was mostly termed ‘reflux’. A ‘burning sensation in the chest’ was considered by many participants in the UK and USA to be heartburn, but it was less certainly identified as such in Brazil and Russia. It seems inevitable that national differences in vocabulary and symptom interpretation will, to some degree, extend to physicians. Thus, although translation of basic medical terms such as heartburn, reflux and regurgitation into different languages may be straightforward, the words may have different nuanced meanings in different countries. Such differences have received little attention in formal studies, but it is apparent that linguistic and cultural factors will influence a patient’s understanding of his/her symptoms.

The use of market research panels does not allow direct comparison with studies of unselected populations or with patients consulting physicians. Moreover, the low threshold of symptom frequency needed to enter our study may also mean that the findings will differ from those reported in many publications. However, difficulty in matching some patients’ descriptions of their symptoms with the conventional medical vocabulary is recognized by most clinicians. When developing questionnaires for self-administration, this potential difficulty has usually been addressed by devising symptom enquiry in a manner that aims to optimize identification of the medically recognized symptoms 4–7,26,27. Typically, patients are provided with symptom descriptions, sometimes supported by word pictures, and it is assumed that the established medical vocabulary can properly represent the patients’ symptoms.

Any suggestion that the established upper GI symptom terminology is flawed may be unsettling for physicians, not least because it may introduce uncertainty into clinical practice. However, our findings serve to endorse the well-accepted clinical principles of listening carefully to patients’ own descriptions of their symptoms and avoidance of putting words into their mouths. The findings also raise some important questions that need direct study. For example, does the conventional concept of heartburn actually embrace two symptoms (‘burning sensation or burning pain in the chest’ and ‘sharp rising pain in the food pipe’) that individuals responding to our questionnaire were seemingly able to distinguish? If these are different symptoms, how should each be interpreted? Likewise, should the symptom ‘blocked feeling or sensation that something is caught in the chest’ identified by some of the study participants be considered dysphagia only if impaired swallowing is also perceived? If there is no swallowing impairment, what should this symptom be called and how should it be interpreted?

Our survey questionnaire was not designed as a tool for clinical practice, nor was it designed to measure symptom burden. No suggestion has been made that it would be suitable for either purpose. Rather, the study has demonstrated that the established medical terminology does not identify some commonly occurring upper GI symptoms that sufferers recognize when described using vocabulary generated by fellow sufferers. Symptom descriptions based on the vocabulary of individuals who suffer them merit closer attention with a view to characterizing upper GI symptoms more precisely.

## Supplementary Material

SUPPLEMENTARY MATERIAL
