# Supplementary material for: Discrepancies between upper GI symptoms described by those who have them and their identification by conventional medical terminology: a survey of sufferers in four countries
Source: Eur J Gastroenterol Hepatol. 2016 Mar 9;28(4):455–62. doi: 10.1097/MEG.0000000000000565 (PMC4777225; doi:10.1097/MEG.0000000000000565)
Supplement: SUPPLEMENTARY MATERIAL [file meg-28-455-s001.pdf]

Supplemental Digital Content 1: On-line Diary Quesions

Subjects were asked to complete the electronic on-line diary daily for up to 42 days. The questions are given below.

SECTION A

Below you will find a diagram of the upper body. Please indicate whether you have experienced today any of the following symptoms in the areas of your body labelled A, C, F, H, I or J.

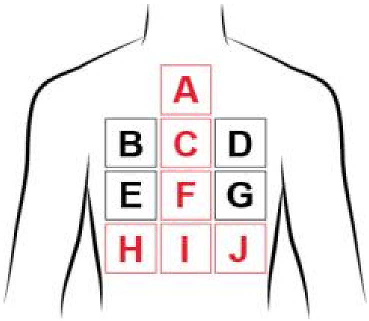

PLEASE WRITE IN THE LOCATION LETTER FOR ALL THAT APPLY IN THE APPROPRIATE COLUMN FOR TODAY (i.e. if this is the first day then use the column labelled "Day 1").

NONE OF THESE SYMPTOMS TODAY (put tick in this row)

Bitter or acidic taste in the (back of) throat / mouth
Sharp rising pain in the food pipe
Burning sensation or burning pain in chest (not related to heart problems)
Burning sensation or burning pain in middle of abdomen (above belly button but not in chest)
Burning sensation or burning pain in stomach
Blocked feeling or sensation that something caught - in chest
Dull ache or discomfort (non-burning) in middle of abdomen (above belly button but not in chest)
Dull ache or discomfort (non-burning) in stomach
Uncomfortably full / heavy stomach after regular-sized meal

IF YOU HAVE EXPERIENCED ANY OF THESE SYMPTOMS IN THE AREAS OF YOUR BODY LABELLED A, C, F, H, I or J THEN PLEASE ANSWER SECTION B TODAY. IF YOU HAVE NOT EXPERIENCED ANY OF THESE SYMPTOMS TODAY THEN YOU DO NOT HAVE TO ANSWER SECTION B TODAY - START WITH SECTION A AGAIN TOMORROW.

SECTION B

1 Please tick the month and date for today

2 What day of the week is it today?

Monday
Tuesday
Wednesday
Thursday
Friday
Saturday
Sunday

3 From the following symptoms - which one of these was the predominant/most intense symptom you experienced today?

1. Bitter or acidic taste in the (back of) throat / mouth
2. Sharp rising pain in the food pipe
3. Burning sensation or burning pain in chest (not related to heart problems)
4. Burning sensation or burning pain in middle of abdomen (above belly button but not in chest)
5. Burning sensation or burning pain in stomach
6. Blocked feeling or sensation that something caught - in chest
7. Dull ache or discomfort (non-burning) in middle of abdomen (above belly button but not in chest)
8. Dull ache or discomfort (non-burning) in stomach
9. Uncomfortably full / heavy stomach after regular-sized meal

4 Thinking of this predominant/most intense symptom, how strong were these symptoms today?

1. Severe
2. Average
3. Mild

5 At what time were these symptoms particularly strong?

1. In the morning when I woke up
2. In the morning after breakfast
3. In the morning (other)
4. In the afternoon after lunch
5. In the afternoon (other)
6. In the evening, after dinner
7. In the evening (other)
8. Before going to bed

9. During the night
10. All day long

6. How long in total did your symptoms last on this occasion?

1. More than 8 hours
2. About 4–8 hours
3. About 2–4 hours
4. About 1–2 hours
5. Less than an hour (but more than 15 minutes).
6. Less than 15 minutes
