# Supplementary material for: Discrepancies between upper GI symptoms described by those who have them and their identification by conventional medical terminology: a survey of sufferers in four countries
Source: Eur J Gastroenterol Hepatol. 2016 Mar 9;28(4):455–62. doi: 10.1097/MEG.0000000000000565 (PMC4777225; doi:10.1097/MEG.0000000000000565)
Supplement: SUPPLEMENTARY MATERIAL [file meg-28-455-s002.pdf]

Supplemental Digital Content 2: Frequency of symptoms identified by respondents as predominant

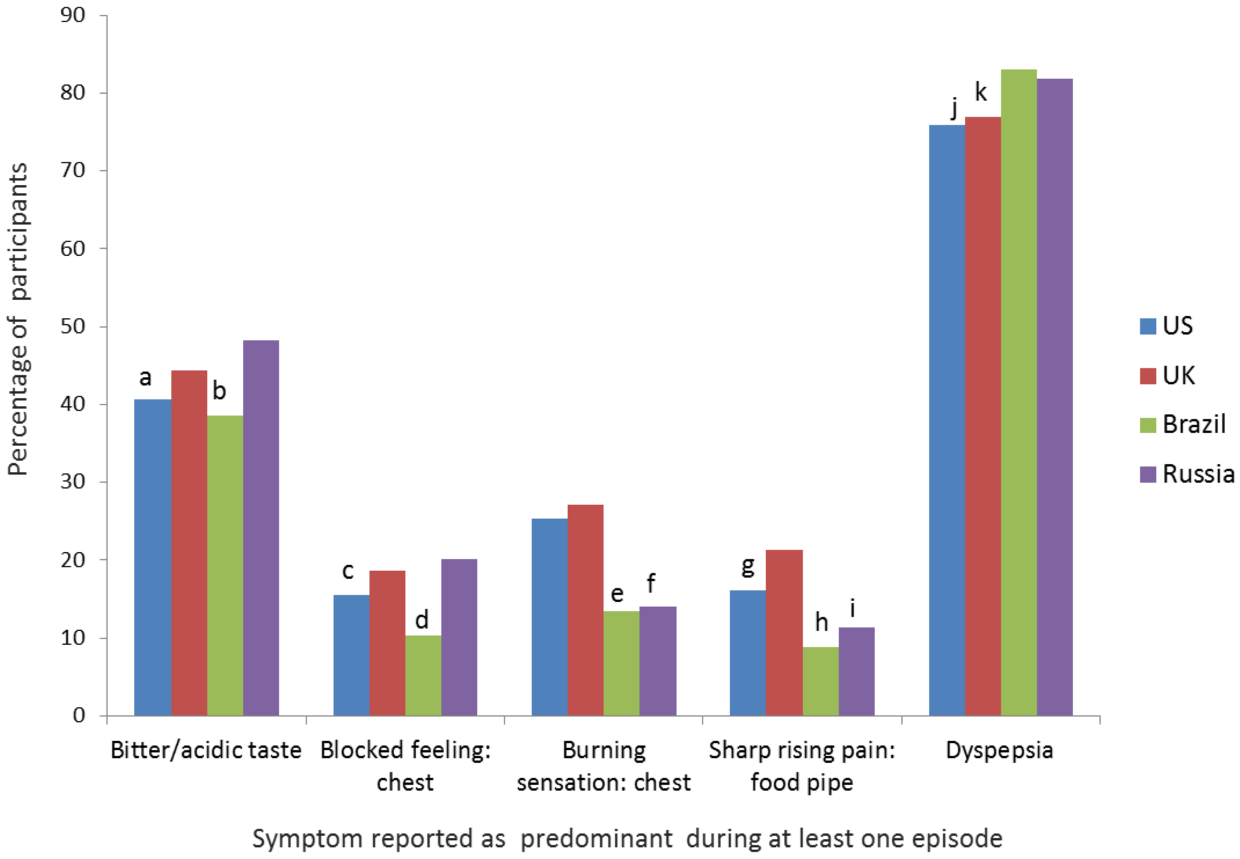

Pairwise comparisons: [a]*p*=0.006 vs. Russia, [b]*p*=0.040 vs. UK; *p*=0.0004 vs. Russia; [c]*p*=0.031 vs. Russia; [d]*p*=0.008 vs. USA, *p*<0.0001 vs. UK and Russia; [e]*p*<0.0001 vs. USA and UK; [f]*p*<0.0001 vs. USA and UK; [g]*p*=0.020 vs. UK; [h]*p*=0.0003 vs. USA and *p*<0.0001 vs. UK; [i]*p*=0.012 vs. USA and *p*<0.0001 vs. UK; [j]*p*=0.0028 vs. Brazil and *p*=0.0062 vs. Russia; [k]*p*=0.0078 vs. Brazil and *p*=0.018 vs. Russia.
