# Supplementary figures and images for: Discrepancies between upper GI symptoms described by those who have them and their identification by conventional medical terminology: a survey of sufferers in four countries
Source: Eur J Gastroenterol Hepatol. 2016 Mar 9;28(4):455–62. doi: 10.1097/MEG.0000000000000565 (PMC4777225; doi:10.1097/MEG.0000000000000565)

### Supplemental Digital Content 3: Duration of predominant symptom by country

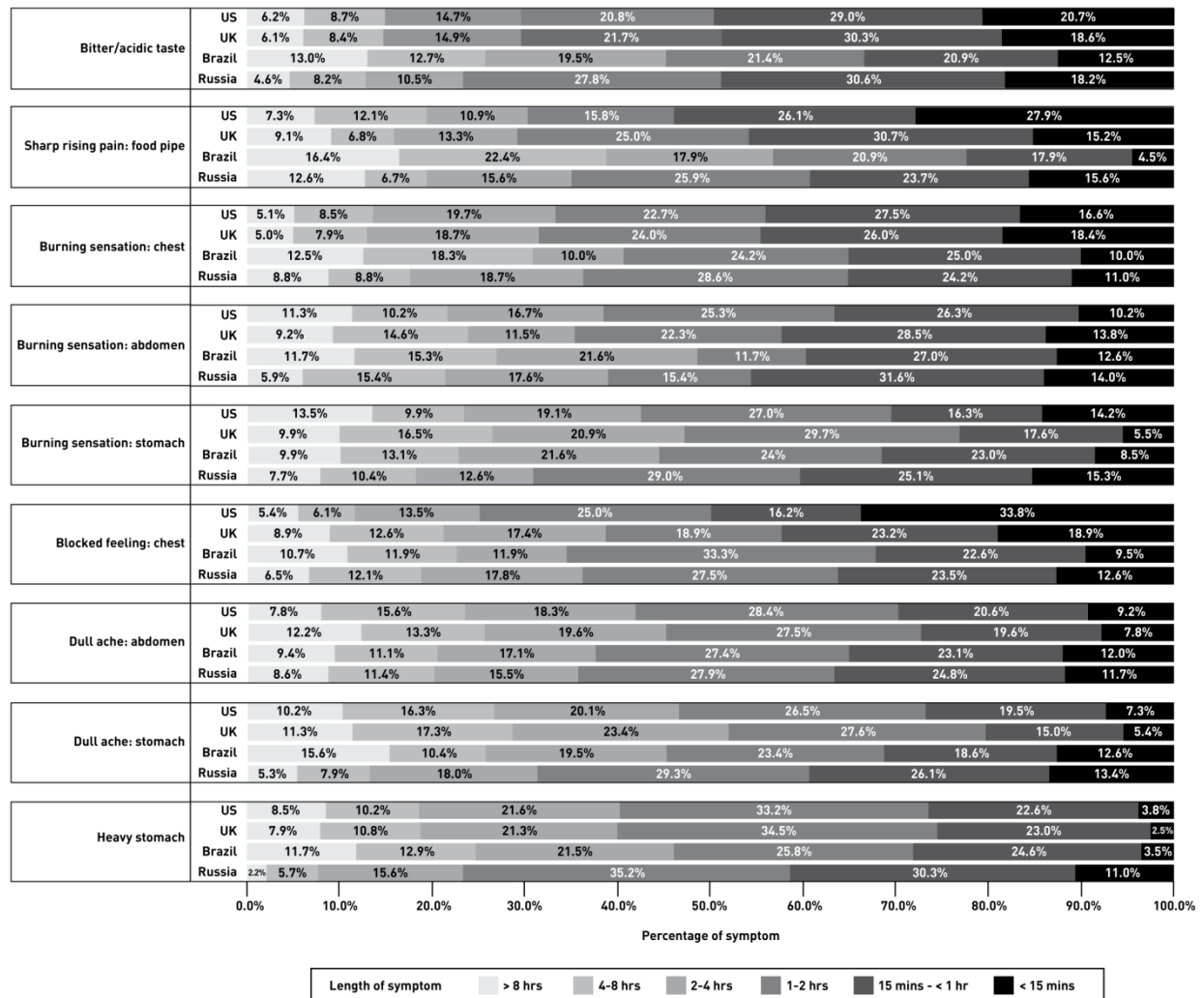

Supplement: SUPPLEMENTARY MATERIAL [file meg-28-455-s003.pdf]

## Supplemental Digital Content 4: Duration of predominant symptom by age

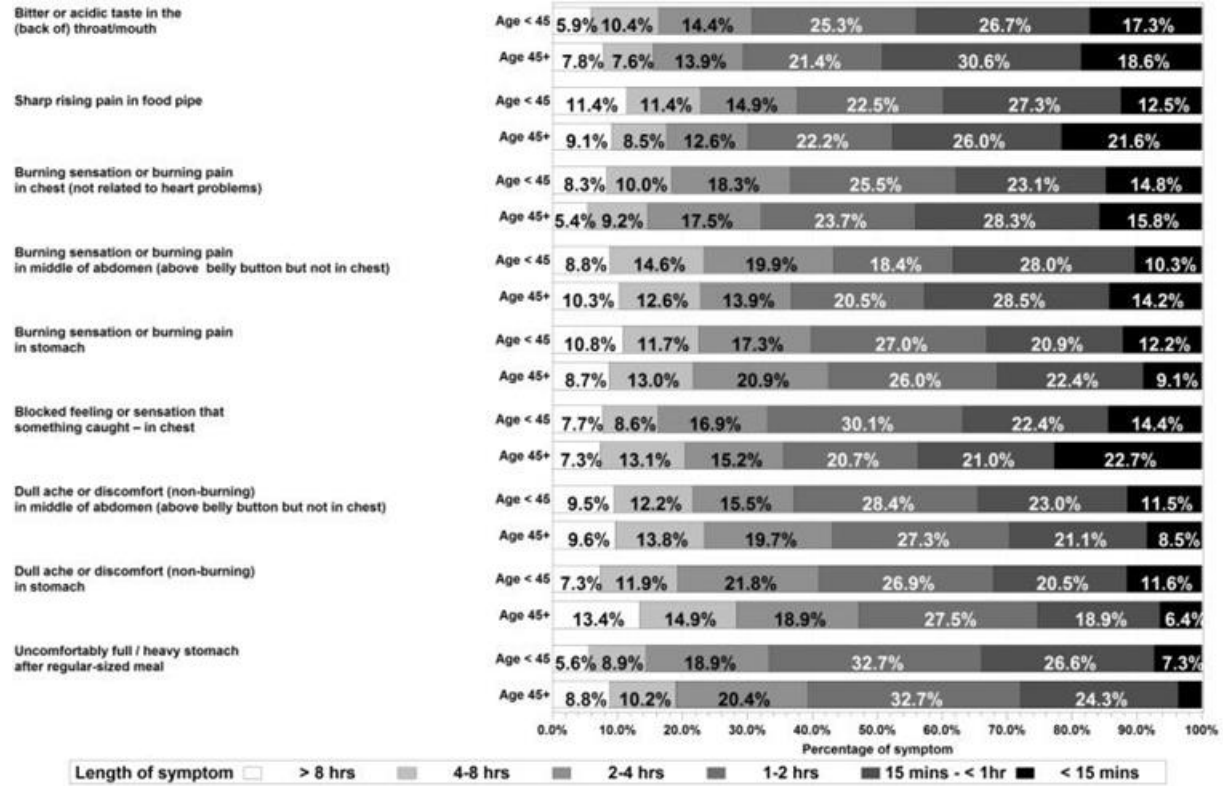

Supplement: SUPPLEMENTARY MATERIAL [file meg-28-455-s004.pdf]

## Supplemental Digital Content 5: Duration of predominant symptom by gender

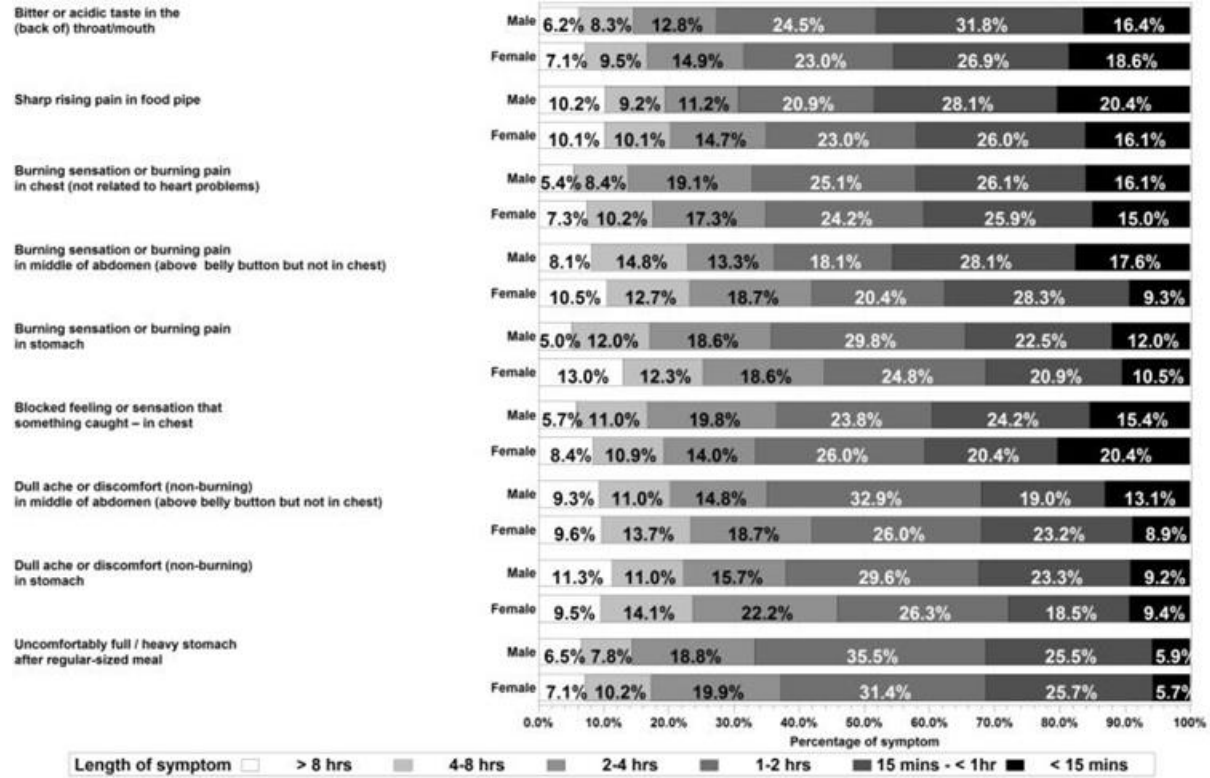

Supplement: SUPPLEMENTARY MATERIAL [file meg-28-455-s005.pdf]
